# Role of different *Culicoides* vectors (Diptera: Ceratopogonidae) in bluetongue virus transmission and overwintering in Sardinia (Italy)

**DOI:** 10.1186/s13071-016-1733-9

**Published:** 2016-08-09

**Authors:** Cipriano Foxi, Gavino Delrio, Giovanni Falchi, Maria Giovanna Marche, Giuseppe Satta, Luca Ruiu

**Affiliations:** 1 Dipartimento di Agraria, University of Sassari, Via E. De Nicola, Sassari, Italy; 2 Istituto Zooprofilattico Sperimentale della Sardegna, Via Duca degli Abruzzi 8, Sassari, Italy

**Keywords:** Bluetongue, BTV, Virus detection, Virus overwintering, Biting midges, *Culicoides* vectors, Seasonal abundance, Infection rate

## Abstract

**Background:**

Bluetongue (BT) epidemics have affected the Mediterranean island of Sardinia since 2000. While *Culicoides imicola* represents the main bluetongue virus (BTV) vector, other European *Culicoides* biting midges, possibly implicated in virus transmission, have been detected here. Understanding their distribution, seasonal abundance, and infection rates is necessary to predict disease incidence and spread across coastal and inland areas, and to define their role in virus overwintering.

**Methods:**

Biting midge abundance was determined by light traps on selected farms representing diverse climatic conditions of Sardinia. Livestock-associated *Culicoides* species were morphologically and molecularly identified. Infection rates in prevailing midge species captured in 2013 during a BTV-1 outbreak were determined using RT-qPCR based virus detection in insect body pools, supplemented by specific body region analyses. The seasonal infection prevalence in *Culicoides* samples collected in 2001 in a BTV-2 affected farm was also determined.

**Results:**

The Newsteadi complex (*C. newsteadi* species A and species B) prevailed among all biting midge species (47.7 %), followed by *C. imicola* (27.8 %) and the Obsoletus complex (*C. obsoletus* and *C. scoticus*) (17.6 %). Whilst *Culicoides imicola* was more abundant along the coast, the Newsteadi complex was frequently collected at higher altitude and the Obsoletus complex was notably associated to cattle farms. *Culicoides pulicaris* and *C. punctatus* abundance was found to be marginal in all farms. BTV was detected in parous female samples of all these species, and the full dissemination of the virus within the body of *C. imicola*, *C. obsoletus*, *C. scoticus*, and Newsteadi complex species was confirmed by analyses of thorax and head, containing salivary glands. Higher infection rates were associated with *C. scoticus*, *C. newsteadi* species A and species B, compared to *C. imicola*. The virus was detected in *C. newsteadi* species A and *C. obsoletus* in winter and spring, whereas it was mainly found in summer and autumn in *C. imicola*.

**Conclusions:**

In Sardinia, bluetongue virus is transmitted by multiple *Culicoides* vectors, including *C. imicola* and the Newsteadi complex being the most important. The Newsteadi complex and other midge species can play an important role in internal areas and are likely to be directly involved in virus overwintering.

## Background

With nearly 1,350 worldwide distributed species (at least 117 in Europe), the genus *Culicoides* (Diptera: Ceratopogonidae) is characterized by a significant diversity of biting midges [1] whose hematophagous females can transmit a variety of filarial worms, protozoans and arthropod-borne viruses to man and wild or domestic animals [2]. Bluetongue virus (BTV), which includes at least 27 different serotypes so far identified [3, 4], is the etiological agent of the internationally significant bluetongue (BT) disease that affects feral and farmed ruminants, and is among the viruses transmitted by these insect pests of medical and veterinary importance in Europe [5].

Insect vectors play a main role in BTV circulation among ruminants, although minor events of host to host transmission are possible through secondary routes including transplacental, iatrogenic and direct contact mechanisms. [6] BTV transmission is mainly carried out by *Culicoides* biting midges that acquire the virus through ingestion of blood from viraemic vertebrate hosts [2]. After infecting susceptible *Culicoides* females, the arbovirus replicates in midgut cells, reaches the salivary glands and is consequently transmitted through further biting activities on animal hosts [7]. Only parous females that have completed a gonotrophic cycle are able to transmit the virus via subsequent blood meals, over their remaining life span. Vector susceptibility and BTV infection are genetically heritable traits that depend on several factors, such as *Culicoides* species/population and virus strain [8], but it is also influenced by extrinsic factors such as temperature [9]. Despite the huge diversity within the genus *Culicoides*, only around 30 species have been associated with BTV transmission [10].

BTV vector competence was demonstrated by laboratory infection studies only for few *Culicoides* species including *C. sonorensis*, *C. imicola*, *C. obsoletus* and *C. scoticus* [11]. However, vector competence can also be evaluated through virus detection and quantification in field-collected *Culicoides* midges. In fact, the direct detection of BTV in the head and thorax, due to the lack of salivary gland infection barriers, could indicate successful virus dissemination in the body of these insects [12]. Moreover, evidence of virus replication in parous females can also be demonstrated by the detection of high virus genome load in their body [13]. In line with this approach, *Culicoides* and BTV diagnostic technologies based on modern Real-time Quantitative Reverse Transcription-Polymerase Chain Reaction (RT-qPCR) may allow the separation of individuals with sub-transmissible infections from the fully disseminated ones, as demonstrated in laboratory assays with *C. sonorensis* [14]. In Europe, potential vector species were identified from studies based on virus isolation or detection by RT-qPCR in field-collected parous females [15–21], detection of virus dissemination in field individuals [13], and arboviral infection in laboratory assays [22–24]. Consequently, *C. imicola*, *C. obsoletus* and *C. scoticus* are presently considered as confirmed BTV vectors, while *C. chiopterus*, *C. dewulfi*, *C. pulicaris* and *C. punctatus* as probable vectors [25].

Since 2000, Sardinia, an Italian island located in the middle of the Mediterranean basin, has been affected by BTV serotypes 1, 2, 4, 8 and 16. The most severe epidemics occurred in 2000–2001 (BTV-2) and in 2013–2014 (BTV-1 and 4), resulting in the loss of more than 500,000 [26] and 100,000 sheep [27] of an approximately 3.5 million total population, respectively. These two main epidemics persisted for two consecutive years, demonstrating the ability of the virus to overwinter in this environment.

In Sardinia, *Culicoides* fauna comprises at least 45 species, of which only few are mammalophilic and livestock-associated. These include the confirmed BTV vectors, *C. imicola*, *C. obsoletus* and *C. scoticus*, that belong to the subgenus *Avaritia,* and the probable vectors *C. pulicaris* (*s.l*.), *C. punctatus* and two genetically distinct species of the Newsteadi complex, included within the subgenus *Culicoides* [28, 29]. BTV-1 was detected by RT-qPCR in field-collected parous females of all these species during 2012–2013 epidemics [30]. Another larger study, conducted in different Italian regions during BTV-1 and BTV-4 outbreaks in 2012–2014, confirmed virus positivity for the same vector species and, additionally, for *C. montanus* and *C. dewulfi*. Remarkably, high virus genome loads were detected in pools of *C. newsteadi* (*s.l*.) from southern Italy, which substantiates the potential role of this species in transmitting BTV during these outbreaks [31].

Based on this information, it is clear that there are different vectors potentially contributing to BTV transmission in Europe. However, for the successful spread of the disease, a vector species not only has to be virus-competent, but it should also be significantly abundant along with an adequate biting rate and host preference within a specific ecosystem [32]. However, information on the actual vector capacity of *Culicoides* species are still insufficient and specific studies on their distribution and abundance in diverse environments characterized by BTV epidemics are needed. In addition, virus overwintering mechanisms in different areas of the world still need to be elucidated. Recent studies from Sardinia reported high abundance of C. *obsoletus*, *C. scoticus*, and *C. newsteadi* (*s.l*.) populations in winter (and not *C. imicola*), implicating these species [28, 33].

To help close this gap, we studied distribution, abundance and seasonality of *Culicoides* species involved in BTV transmission in relation to climatic factors and the availability of blood meal sources. These investigations included RT-qPCR-based virus detection and infection rate determination in field-collected midge species from livestock farms affected by BTV epidemics in 2012–2013 and, retrospectively, in 2000–2001.

Through comparative analyses of different *Culicoides* species distribution, seasonal abundance, and infection rates, we examined their actual role in transmission and overwintering of the virus in coastal and internal areas of Sardinia, which generated awareness on the dynamics of potential vectors in a specific ecosystem. The scalability of this study model to a variety of Mediterranean environments will allow implementing more appropriate disease risk analyses and effective integrated vector management measures.

## Methods

### Study area

Studies were conducted in Sardinia, a region with a typical Mediterranean climate where winter is mild with rare freezing temperatures along the coast and summer is hot and dry with day temperatures often exceeding 30 °C. Although the inland summer temperatures are high, the winter temperatures frequently drop below zero degrees. The island receives most rainfall during October-April, on an average 400–600 mm along the coast and 500–800 mm inland, respectively per year.

A total of ten farms, representing different climatic conditions of Sardinia and differently stocked with animals, were involved in this study (Table 1).

**Table 1:** Characteristics of the 10 farms where *Culicoides* midges were collected by light traps in Sardinia

Collection sites	Geographic coordinates (sites ranked North to South)	Altitude (m asl)	Mean low temperature of the coldest month (°C)	Mean high temperature of the warmest month (°C)	Annual precipitation (mm)	Host diversity and host abundance	Trap localisation	Main larval habitats
Arzachena	41°09′N; 9°23′E	77	3.60	33.10	800	200 sheep	outside sheep shelter, close to sheep	leaking watering troughs, permanent puddles, water spring
Sassari-1	40°45′N; 8°12′E	120	5.16	30.70	460	500 sheep	inside sheep shelter, widely open	cattle-watering pond, shallow pool, leaking watering troughs, manure
Sassari-2	40°44′N; 8°12′E	140	5.23	31.80	600	50 cattle; 10 horses; 300 sheep	inside sheep shelter, widely open	cattle-watering pond, shallow pool, manure
Olmedo	40°38′N; 8°22′E	30	2.13	31.37	600	250 sheep	inside sheep shelter, widely open	natural drainage channel, leaking watering troughs
Fonni	40°07′N; 9°18′E	980	-2.66	32.33	810	100 sheep	outside sheep shelter, close to sheep	natural drainage channel, sheep-watering pond
Seneghe	40°03′N; 8°32′E	160	5.90	34.00	500	60 cattle; 250 sheep	inside cattle barn, with little openings	leaking watering troughs, natural drainage channel, manure
Barisardo	39°81′N’ 9°65′E	50	3.73	31.03	650	300 sheep	outside sheep shelter, close to sheep	leaking watering troughs
Lanusei	39°52′N; 9°33′E	490	1.00	30.83	500	100 goats	outside, closed to goats	leaking watering troughs
Villaverde	39°48′N; 8°47′E	450	2.85	33.30	500	350 sheep	inside sheep shelter, widely open	natural drainage channel, leaking watering troughs, sheep-watering pond
S. Antioco	38°59′N; 8°25′E	40	7.93	33.13	500	50 cattle	inside cattle barn, widely open	leaking watering troughs, manure

### *Culicoides* midge collection

Adult *Culicoides* were collected using suction light traps (miniature blacklight trap; GZ International, Ferrara, Italy) fitted with a blacklight tube (Philips TL 4 W/08) and a downdraught suction motor. Traps, operating overnight from dusk until dawn of the following day, were hung 1.8 m above the ground inside an open shelter in six farms and outside a shelter close to the livestock in the other four farms.

Trapping was carried out once a week in Sassari-1 during 2001, in Fonni during 2003, and at other seven sites during 2008. In the case of Bari Sardo, weekly captures were limited to the period April-July 2013, during a major BTV outbreak.

### Identification of *Culicoides* midge species

Biting midges were initially identified to species or complex level according to wing pigmentation patterns of both sexes and male genital morphology, using different keys [34–36]. Additional morphometric and molecular analyses were conducted for females of Obsoletus, Pulicaris, and Newsteadi complexes, to differentiate known species.

For Obsoletus complex identification, including *C. obsoletus* (*s.str*.) and *C. scoticus*, the terminal half part of female abdomen was dissected and mounted on glass slides for microscopic observations. The remaining insect body portion was used for molecular analysis based on ITS2 rDNA amplification and sequencing [37] and on species-specific PCR assays targeting COI gene [38]. The following morphometric characters of the abdomen were measured: length between the chitinous plates, length and width of larger and smaller spermathecae [39, 40]. All specimens that were submitted to viral detection assays were also identified by molecular analysis, while representative samples (10–20 %) of females, collected monthly from 6 sites, were identified by morphometric analysis, in order to derive the proportion of *C. obsoletus* and *C. scoticus* in each farm, thus allowing significant cost savings.

Since sibling species belonging to the Pulicaris complex were recently identified through molecular analysis [29, 41–44], after being morphologically identified, *C. pulicaris* (*s.l*.) samples were submitted to species-specific PCR assays based on mitochondrial cytochrome *c * oxidase subunit I (COI) gene amplification [38].

To identify species belonging to the Newsteadi complex, both molecular and morphological analysis were conducted. Midges were preliminarily distinguished into two forms based on wing pattern and additional morphometric characters (i.e. separation of eyes and palpal segment size): (i) form A, with wider and less pigmented wings showing small and diffused pale spots at the tip of vein M_1_ and M_2_; and (ii) form B, with smaller and more pigmented wings with well-defined, clear pale spots at the tip of M_1_, M_2_ and C_1_ veins. Subsequent analyses of the ribosomal internal transcribed spacer 2 (ITS2 rDNA) sequences were performed to confirm the reliability of these morphological characters to discriminate between *C. newsteadi* species A and B, as previously detected in Sardinia [29]. Recent morphometric and molecular investigations on the Newsteadi complex have led to the identification of different species in diverse geographical areas of Europe [29, 38, 41, 42, 44, 45]. To verify correlation between *C. newsteadi* N1, N2, and N3 from Catalonia, Spain [41] and *C. newsteadi* species A and B, we analyzed our samples with species-specific PCR assays targeting COI gene.

The females of all species were age-graded according to the abdominal pigmentation [46] and small pools (≤ 10 specimens) of parous females, after identification procedures, were kept in ethanol (70 %) at 4 ° C until being used for BTV detection analyses.

### Detection of BTV infection of *Culicoides* midges

Analyses for BTV detection were based on total RNA extraction from pools of parous females belonging to different *Culicoides* species from the same site and date of capture, followed by retro-transcription to cDNA and qPCR with serotype-specific primers targeting BTV genome segment 2 [3].

Insect pools were routinely washed in PBS and transferred into 1.5 ml Eppendorf tubes containing 700 ul Trizol® Reagent (Life Technologies, USA) and 200 μl of 1-mm-diameter zirconia beads, and homogenized in a Tissue Lyser LT (Qiagen, Hilden, Germany) [47]. cDNA was synthesized using SuperScript™ II Reverse Transcriptase (RT) (Life Technologies, USA) in compliance with the manufacturer’s instructions. Subsequently, cDNA was amplified by PCR with 1A2 for BTV-1 and 2A1 for BTV-2 primer pairs under conditions described in Maan et al. [3]. Samples were provisionally considered positive with the virus when an amplified product of the predicted size was observed on an agarose gel. For confirmation, some of the bands of interest were excised from the gel and amplicons purified using a QIAquick gel extraction kit (Qiagen, Hilden, Germany) for sequencing. Output sequences were analyzed by NCBI Basic Local Alignment Search Tool http://www.ncbi.nlm.nih.gov/blast/Blast.cgi. For quantitative purposes, all positive samples were further analyzed by real-time RT-qPCR assay using the SuperScript™ III One-Step RT-PCR System with Platinum® Taq High Fidelity in compliance with the manufacturer’s instructions and in accordance with a standardized assay [48]. Amplification was carried out in an Applied Biosystems 7900HT Fast Real-Time PCR System and fluorescence was measured at the end of the 60 °C annealing/extension step. Cycle threshold (Ct) values for each sample were determined when a threshold fluorescence line was breached. Insect pools with a cycle threshold (Ct) value < 30 were confirmed as BTV positive.

*Culicoides* species involved in virus detection analyses were collected during spring and summer 2013 in a BTV-1 affected sheep farm located in Bari Sardo [49]. This investigation involved 1,622 parous females grouped into 163 species-specific pools including all the livestock-associated *Culicoides* species in Sardinia. According to preliminary studies demonstrating an efficient detection of the virus by RT-qPCR on a single infected midge, pool size was limited to 10. All analyses were generally conducted on the full insect body, except for *C. obsoletus* and *C. scoticus* whose terminal part of abdomen was previously employed for identification purposes.

To confirm the full dissemination of the virus in BTV positive species, additional RT-qPCR analyses were conducted to detect the virus in head and thorax regions of small pools (5 individuals) of parous females of *C. imicola, C. obsoletus, C. scoticus* and *C. newsteadi* species A and B. The remaining abdominal region was used for genetic discriminations among species of Obsoletus and Newsteadi complexes. *Culicoides pulicaris* (*s.str.*) and *C. punctatus* were not included in these analyses because of the lack of a sufficient number of positive specimens.

A retrospective BTV infection prevalence survey was also conducted on ethanol-stored *Culicoides* midges captured in the Sassari-1 farm, during a BTV-2 outbreak in 2001. This investigation had the specific purpose of establishing virus prevalence in *Culicoides* species over the year. Analyses were carried out on monthly collected parous females grouped in 68 species-specific pools of *C. imicola*, *C. obsoletus* and *C. newsteadi* species A and B.

### Statistical analysis

Association between *Culicoides* species abundance and collection sites in 2008 was assessed by Pearson’s Chi-square (*χ*^2^) test using R software with significance level set at α = 0.05 [50].

The minimum infection rates (expressed per 1,000 parous females) in prevailing midge species collected during BTV-1 outbreak in 2013 were calculated using PooledInfRate software [51].

## Results

### Distribution, abundance and seasonality of livestock-associated *Culicoides* midges

**Fig. 1:**
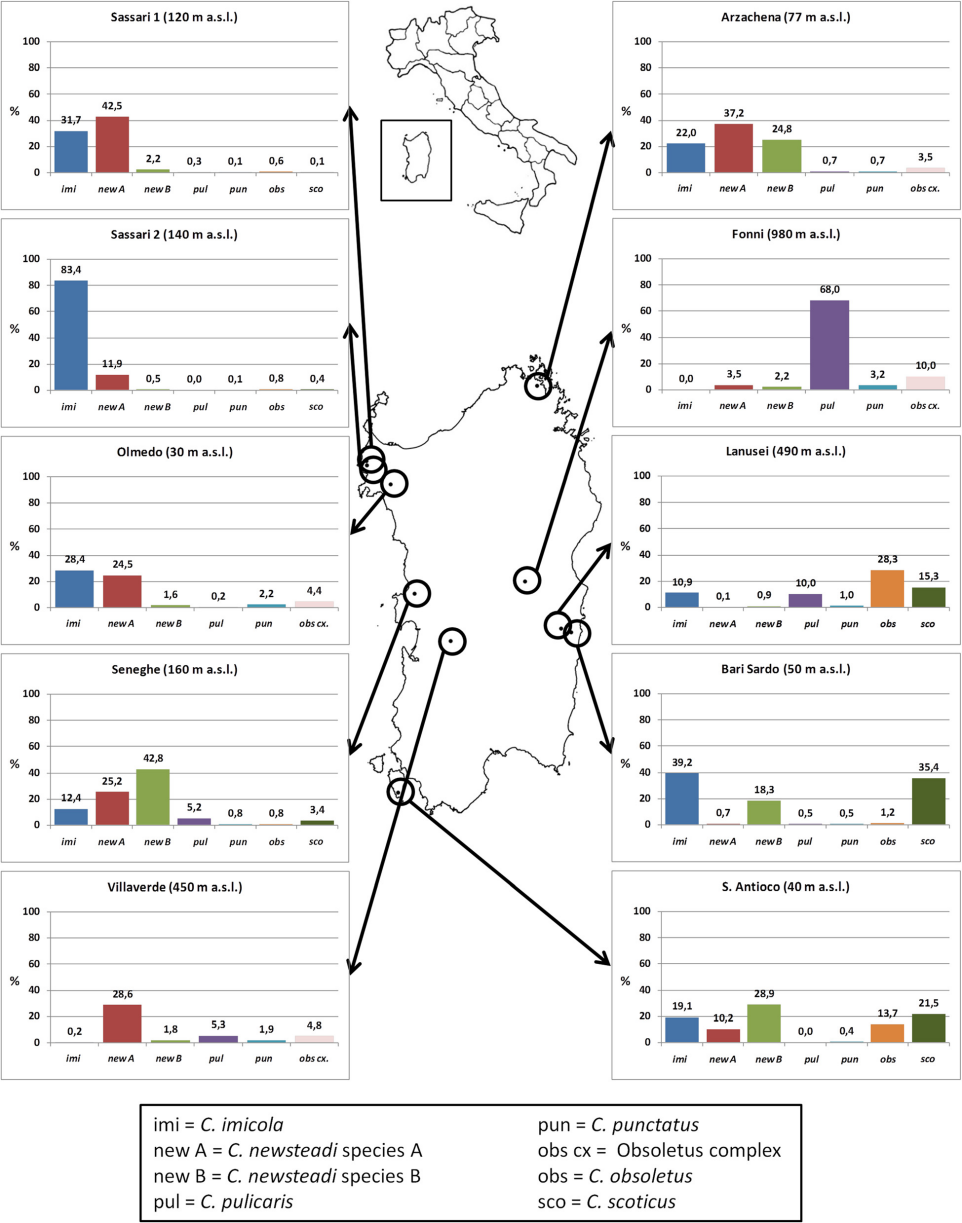
Map of Sardinia showing the location of study farms and the frequency of livestock-associated *Culicoides* species

A total of 140,624 *Culicoides* adults (131,783 females and 8,841 males) belonging to 15 different species were collected during 441 survey nights by traps placed in ten different livestock farms in Sardinia. All farms included diverse *Culicoides* larval habitats that were mostly associated with leaking drinking troughs, watering ponds, natural drainage channels, and manure heaps (Table 1). The majority of midge adults (134,305 specimens) represented species normally associated with animals and known to be proven or potential BTV vectors in the Mediterranean region: *C. imicola*, Obsoletus complex (*C. obsoletus* and *C. scoticus)*, *C. pulicaris* (*s.l*.), *C. punctatus*, and Newsteadi complex (*C. newsteadi* species A and B). With the sole exception of *C. imicola* that was not found in the farm at higher altitude, all the other species were detected in varying proportions in each farm (Table 2 and Fig. 1), with a significant dependence between species and site of collection (Pearson’s Chi-square test: *χ*^*2*^ = 56767.59, *df* = 24, *P* < 0.001).

**Table 2:** Relative numbers of *Culicoides* species collected one night per week by light traps in Sardinia

Collection sites	Year	Total *Culicoides*	*C. imicola*	Obsoletus complex	*C. obsoletus*	*C. scoticus*	*C. newsteadi* species A	*C. newsteadi* species B	*C. pulicaris* (*s.l.*)	*C. punctatus*
Arzachena	2008	25506	5615	892^b^	–	–	9479	6320	175	184
Sassari 1	2001	12356	3919	87	74	13	5255	277	35	13
Sassari 2	2008	11166	9317	140	94	46	1327	58	4	7
Olmedo	2008	8204	2334	363^b^	–	–	2011	132	15	181
Fonni	2003	8919	0	895^b^	–	–	316	193	6062	287
Seneghe	2008	33548	4150	1433	281	1152	8467	14350	1728	277
Bari Sardo^a^	2013	15162	5948	5559	185	5374	103	2776	77	77
Lanusei	2008	4819	525	2102	1366	736	6	42	480	46
Villaverde	2008	3761	6	182^b^	–	–	1075	69	201	71
S. Antioco	2008	17183	3286	6053	2361	3692	1759	4958	4	69

^a^Captures between April-July

^b^Obsoletus complex species were not distinguished

The Newsteadi complex prevailed over other midge species, representing 47.7 % of the whole population of potential vectors collected during the study period in different farms. Within this complex, *C. newsteadi* species A and B, identified through molecular analysis, were reliably distinguished also by wing pattern examination. Species-specific PCR assays targeting COI gene, showed a significant correspondence between *C. newsteadi* species A from Sardinia and N1 from Catalonia [41]. *Culicoides newsteadi* species A and B were both detected in every farm, with varying ratios. In detail, species A prevailed (60–96 %) in four farms located at lower altitude in northern Sardinia, while species B was more common (63–96 %) in farms based in the central-southern part of the island, excluding a farm in Villaverde (450 m asl) with just 6 %. *Culicoides imicola* was the second most captured species representing 27.8 % of the whole midge collection. The distribution of this species was remarkably comparable to Newsteadi complex species, showing prevalence in farms along the coast of Sardinia. However, *C. imicola* was never or hardly ever collected in farms above 450 m asl, whereas the Newsteadi complex showed more ecological flexibility, being captured even at 980 m asl. Obsoletus complex species accounted for 17.6 % of total midges, but were the most abundant in three farms where they reached 30 %. Two species of this complex, *C. obsoletus* and *C. scoticus*, were molecularly and morphologically identified. As a result of morphometric measurements on molecularly identified females, the following individual morphological discontinuities were found: larger spermatheca (length range 68–79 and width range 45–55 μm for *C. scoticus*; length range 44–50 and width range 34–38 μm for *C. obsoletus*) and smaller spermatheca (length range 59–77 and width range 44–54 μm for *C. scoticus*; length range 46–49 and width range 34–37 μm for *C. obsoletus*). On the other hand, overlapping was observed for length between the chitinous plates of these species. Both species were reported in six different sampling sites, with general predominance of *C. scoticus*, accounting for 70 % of the whole catch. The abundance of Pulicaris complex, accounting for 7.8 % of total midge collection, was significant only in two farms and it was the predominant species at 980 m asl. Molecular analysis confirmed that the specimens from Bari Sardo used in virus detection analyses were *C. pulicaris* (*s.str*.) [38, 41, 42]. This species was also detected in samples from locations with higher capture (Fonni and Seneghe). However, this molecular confirmation was not available for all samples and further studies on Pulicaris complex in Sardinia are needed. For this reason, *C. pulicaris* (*s.l*.) was used in abundance and distribution data analysis. Besides, *C. punctatus* was detected in all farms, but was the less represented species, amounting to just 1.1 % of total midge captures.

**Fig. 2:**
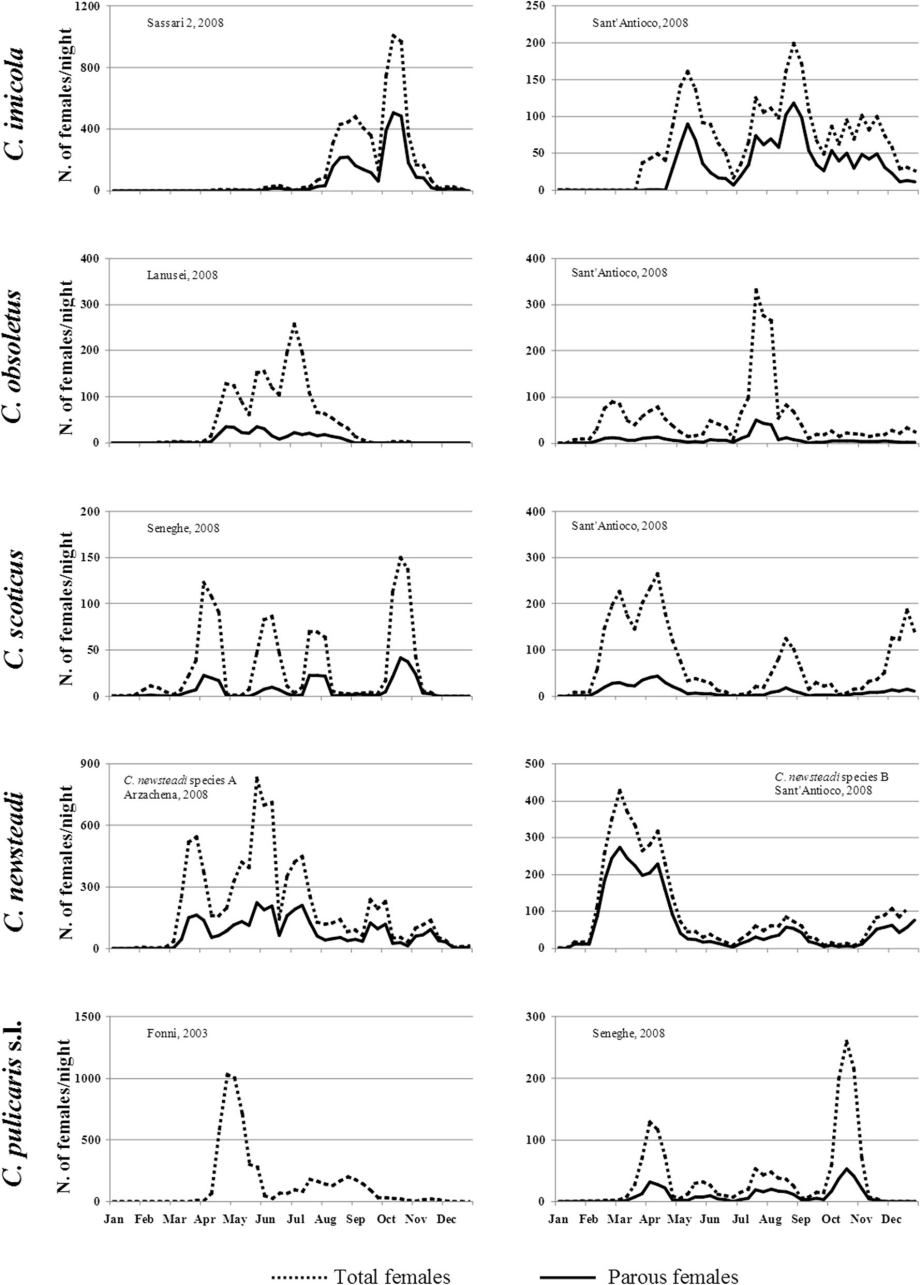
Seasonal abundance pattern of *Culicoides* midges in representative farms selected on the basis of the highest catches of each species and comparing a Northern and a Southern location. Dashed lines represent the nightly number of light-trapped females; solid lines depict the nightly number of parous females

The amount of midges collected by traps in each farm, during different years, allowed to adequately define a detailed seasonal abundance pattern for adults and parous females of six different *Culicoides* species. Seasonal dynamics in farms, representative of a Northern and a Southern location selected on the basis of the highest catches of each species, were obtained by calculating a three-week moving average of the raw count data. This was carried out in order to reduce spurious temporal changes in abundance due to variation in meteorological conditions during the weekly trapping effort (Fig. 2) [52]. The seasonal abundance of *C. newsteadi* species A and B did not differ within the same farm, and adults were captured by light traps throughout the year displaying 3 marked population peaks in spring, summer and autumn (Arzachena, 2008). Generally, the maximum abundance was reached during June-August; however, in the case of the southern farm (Sant’Antioco, 2008), *C. newsteadi* species B was more abundant in February-May. Parous females of the Newsteadi complex were collected throughout the year and represented, on average, 35 % of total females collected, with whom they shared the same seasonal trend. *Culicoides imicola* was never or rarely captured during the first five months of the year, which was followed by a rapid population increase starting from July and reaching the peak in summer-autumn (Sassari-2, 2008). In the southern farm (Sant’Antioco, 2008), an earlier population growth was recorded in April. The number of parous females represented 45 % of total females. *Culicoides scoticus* was detected during the whole year with 3–4 population peaks depending on the farm, and was generally more abundant during the first or the last months of the year (Seneghe and Sant’Antioco, 2008). In the southern farm, parous females collected during February-April accounted for 66.5 % of the entire year collection, and in the same period their number per trap/night ranged between 14 and 71, respectively. Similarly, *C. obsoletus* was detected over the whole year, but the highest density was recorded during summer (Lanusei and Sant’Antioco, 2008). *Culicoides pulicaris* showed four abundance peaks during April-November, while its abundance during the first months of the year was very low (Fonni, 2003; Seneghe, 2008). The majority of adults were collected in April in the 980 m asl site. The abundance peaks for parous females (26 % of total females) were similar to those recorded for the total female population.

### Detection of BTV infection of *Culicoides* midges

BTV-1 was significantly detected in 63 out of 163 species-specific pools of biting midges collected during an outbreak between April and July 2013 in Bari Sardo. Among these, 35 (21.5 %) and 17 (10.4 %) carried a high (Ct range: 25–30) and a very high (Ct < 25) virus genome load, respectively. The higher loads were found in pools of *C. imicola*, *C newsteadi* species A and B, and *C. scoticus* parous females*,* that represented the majority of pools analyzed during this period. *Culicoides obsoletus*, *C. pulicaris* and *C. punctatus,* collected from April to May, were represented in only a few female pools analyzed, but were also found to be BTV-1 positive (Ct range: 25–30) (Table 3).

**Table 3:** RT-qPCR analyses of BTV-1 on *Culicoides* parous females collected in April-July 2013 in Bari Sardo

Species	No. of pools tested	No. of pools with Ct values < 25	No. of pools with Ct values 25-30	No. of pools with Ct values 31-40
*C. imicola*	52	5	6	1
*C. obsoletus*	2	0	1	0
*C. scoticus*	24	4	7	2
*C. newsteadi* species A	46	5	11	5
*C. newsteadi* species B	32	3	6	2
*C. pulicaris* (*s.str.*)	1	0	1	0
*C. punctatus*	6	0	3	1

The results of RT-qPCR for the detection of the virus in specific parous female body regions (head and thorax) confirmed the full dissemination of BTV within the main midge vectors. Higher virus genome loads were found in head and thorax of *C. scoticus* and *C. newsteadi* species A, and in thorax of *C. imicola* and *C. newsteadi* species B (Table 4).

**Table 4:** RT-qPCR analyses targeting BTV-1 in different body regions of *Culicoides* parous females collected in May-July 2013 in Bari Sardo

Species	No. of pools	No. of positive pools	qPCR Ct values	
			Head	Thorax
*C. imicola*	4	1	> 40	24.25
*C. scoticus*	4	2	24.37 > 40	26.15 27.20
*C. newsteadi* species A	4	1	28.13	26.70
*C. newsteadi* species B	4	1	> 40	26.20
*C. obsoletus*	2	1	> 40	25.47

The minimum infection rates (MIRs), expressed per 1,000 parous females, has been calculated for the prevailing midge vectors for which a sufficient number of samples was available. *Culicoides scoticus* had the highest MIR value of 0.058, while the lowest rate was that for *C. imicola* (0.023). *C. newsteadi* species A and species B showed intermediate values (Table 5).

**Table 5:** Minimum Infection Rate (MIR)^a^ in parous females of *Culicoides* vectors from Bari Sardo

Species	Infection Rate	Lower Limit	Upper Limit	No. ofpools	No. ofpositive pools	No. ofindividuals
*C. imicola*	0.023	0.012	0.040	52	11	520
*C. scoticus*	0.058	0.031	0.101	24	11	240
*C. newsteadi* species A	0.041	0.025	0.066	46	16	460
*C. newsteadi* species B	0.032	0.016	0.058	32	9	320

^a^MIR = number of positive pools/total specimens tested, assuming only one infected individual midge per positive pool [51]

### BTV infection prevalence in *Culicoides* vectors over the year

**Fig. 3:**
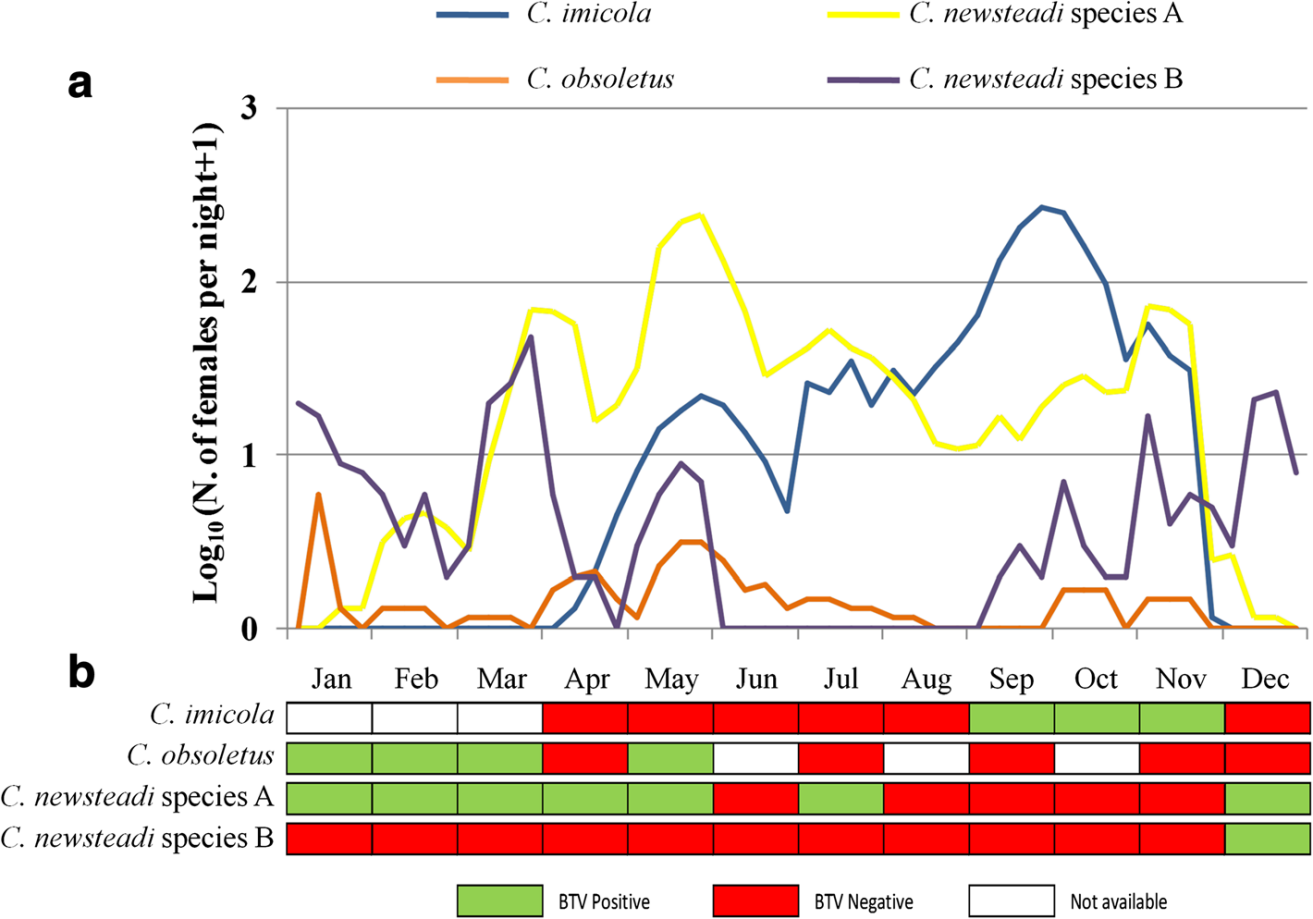
*Culicoides* abundance and BTV prevalence in Sassari-1 during 2001. **a**) seasonal patterns of abundance of parous females and **b**) BTV-2 infection prevalence per month

Retrospective analyses on virus infection were carried out on ethanol-stored parous females of different *Culicoides* vectors, collected monthly, in Sassari-1 farm during a major BTV-2 epidemic in 2001. In this farm, *C. newsteadi* species A (95 % of the Newsteadi complex) was captured throughout the year, with higher abundance in spring, and represented the most prevalent species, followed by *C. imicola*, captured from April and reaching the population peak in September. The Obsoletus complex, showed a lower population density and included mainly *C. obsoletus* (85 %) (Fig. 3).

RNA virus was detected and quantified by RT-qPCR in the insect body of the two Newsteadi complex species, and of *C. imicola* and *C. obsoletus*. BTV-2 positive pools (Ct values < 30) of *C. obsoletus* and *C. newsteadi* species A were detected during the winter-spring months and sporadically in summer, whereas *C. imicola* pools showed virus positivity only from September to November (Fig. 3).

## Discussion

European species of livestock-associated *Culicoides* biting midges involved in BTV transmission and identified in our study include *C. imicola*, *C. obsoletus*, *C. scoticus*, *C. pulicaris*, *C. punctatus* and two species of the Newsteadi complex. Other potential vectors, such as *C. chiopterus* and *C. dewulfi*, were never detected in Sardinia [28]. The Newsteadi complex adults were the most frequent and abundant in the majority of the farms, representing around half the total number of collected midges. The predominance of this cryptic species complex, was observed in previous studies conducted in the two adjacent Mediterranean islands, Sardinia [28] and Corsica [53]. Both *C. newsteadi* species A and B, identified by molecular analysis and wing pattern, were collected in all study farms, but species A generally dominated in Northern Sardinia while species B usually prevailed in the central-southern part of the island (Fig. 1). *Culicoides imicola*, the second most abundant species, was more frequent near the coast while its population density progressively decreased with altitude, due to a relatively poor tolerance to lower temperatures [54]. This is consistent with the well-documented distribution in Sardinia [55, 56] and as described by specific mathematical models [57, 58]. However, its distribution at farm-level can only be predicted by considering the availability of appropriate breeding sites, that are represented by moist soil patches surrounding leaking animal drinking troughs and feces-contaminated pond margins [26]. Furthermore, the normally extremely dry Mediterranean conditions should be taken into consideration, as they may cause a shrinkage in the availability of these anthropogenic breeding sites in summer, as demonstrated by a previous study in northern Sardinia, where *C. imicola* larval foci were found only in 41 of more than 100 sampled potential sites [59]. The *Obsoletus* complex, including *C. obsoletus* (*s.str*.) and *C. scoticus*, represented 17 % of the total *Culicoides* midges collected in Sardinia and was detected in all farms with a general prevalence of *C. scoticus*. The females of this complex were identified by morphometric measures of genitalia combined with molecular analyses. Although measurements of spermathecae of our samples were generally higher in comparison with samples reported in France [39] and other European countries [40], these values did not exhibit overlapping ranges, thus confirming their reliability for the morphometric identification of the two species, allowing a significant laboratory cost saving.

Higher abundance was associated with cow farms along the coast, probably due to the composted cow manure accumulating in cattle holding and spread on cropping lands and pastures. Although *C. obsoletus* and *C. scoticus* are able to breed in a great variety of habitats, dung heaps and cow pats are emerging as their most important farm development substrates in Germany [60]. *Culicoides pulicaris* and *C. punctatus* were generally collected in smaller amounts and a higher abundance of the first species was especially recorded in farms at higher altitudes.

*Culicoides imicola* is a multivoltine species, having several generations per year regulated by temperature and other climatic factors [61]. In Sardinia, adults were collected from April and their density gradually grew to reach a plateau between August and November [28, 62]. For other Palaearctic *Culicoides* vectors, including Obsoletus and Newsteadi complex species that produce 3–4 generations per year [26], parous females were collected from February until the end of the year, with higher density in spring, summer and autumn.

The significant viral RNA titers detected by qRT-PCR in the body of parous females of *C. imicola*, *C. obsoletus*, *C. scoticus* and *C. newsteadi* species A and B, support their vector competence during BTV-2 and BTV-1 epidemics in Sardinia. As a confirmation of the ability of the virus to replicate inside females of these species, meaningful high virus genome loads (Ct values < 25) were found in the thorax, where salivary glands are located, and exceptionally in the head. BTV infection prevalence was also observed in few parous female pools of *C. obsoletus*, *C. pulicaris* and *C. punctatus*, indicating their possible implication in virus transmission in Sardinia. This study confirmed the ability of different *Culicoides* species to harbor both BTV-1 and BTV-2 serotypes, but specific experiments are needed to assess possible differences in their susceptibility levels.

Among BTV-positive midge species collected in this study, *C. imicola*, *C. obsoletus*, and *C. scoticus* are recognized as proven vectors in Europe, as supported by repeated virus isolation from field-collected females [16, 17] and by laboratory infection studies demonstrating their oral susceptibility to the virus [22–24]. *Culicoides newsteadi* has been regarded as a potential vector since few intrathoracically-inoculated females from Israel sustained virus replication in the laboratory [63]. The high virus genome loads we found in thorax and full body samples of *C. newsteadi* species A and B, supported the full BTV dissemination in field-collected parous females, which corroborates the results of other investigations in Italy [31]. While the high amount of viral RNA inside the insect body can be evidence of insect competence, this should be confirmed by the isolation of viable virus particles and proper infection studies in the laboratory. However, Newsteadi complex species should undoubtedly be implicated in virus transmission among animals because of their abundance in internal areas of Sardinia, where *C. imicola* was only sporadically collected. In addition, their winter-spring activity period makes *C. newsteadi* species A and B the main candidates for virus overwintering.

The estimated prevalence of BTV-1 infection in field-collected parous females of most abundant vector species in our study was comparable to previous infection rate determinations in Sardinia and other Italian regions [31]. Higher Minimum Infection Rate (MIR) values were associated to Obsoletus and Newsteadi complex species, indicating a probably higher susceptibility of the Obsoletus complex in comparison to *C. imicola*, which is consistent with laboratory infection studies in England [24]. However, when infection rate calculations are based on light trap collected females, as in our study, a possible underestimation of virus prevalence may result from repellent effects of light against infected vectors [64].

On the other hand, neither the field infection rate determination nor the evaluation of vector competence represent the actual potential of vector populations to transmit the disease among animals. This could more appropriately be estimated through the vector capacity, defined as the average number of infective bites per host per day [65]. Vector capacity depends on either vector competence and various biological traits of the vector such as life history parameters, gonotrophic cycle length, longevity, proportion of blood meals, and host biting rate [32]. Even though the contribution of these parameters is still scarcely investigated on European midge vectors, the actual role of these species during BT epidemics in Sardinia can be evaluated taking into account the main factors affecting the host biting rate, like species distribution, abundance and phenology. Field abundance and host preference of a species can compensate for its limited vector competence and infection rate, as confirmed by studies in Australia where a more important vector role was associated with the relatively inefficient but abundant *C. brevitarsis*, in respect to the more efficient but less abundant *C. fulvus* [66].

In Sardinia, *C. imicola* plays a main role in BTV transmission along coastal areas where this species is significantly abundant, as documented by extraordinary capture levels in certain farms where more than 200,000 midges per night were collected by a single trap. This multivoltine and thermophilic species was rarely captured during the first half of the year and peaked in summer-autumn in conjunction with BT epidemics. Both in the same areas and inland, an augmentative vector role can be attributed to the more abundant *C. newsteadi* species A and B that were found all over the year. An additional contribution to BT spread along the coast and inland may derive from populations of other vectors like *C. obsoletus*, *C. scoticus* and *C. pulicaris*, that can reach high local abundance, especially in cow farms.

The BT epidemic waves in Sardinia normally lasted 2–3 years, and achieved an end as a consequence of natural animal immunization and vaccination campaigns. Infection continuity, over the years, revealed obvious virus overwintering. In this context, parous females of Newsteadi and Obsoletus complex species associated with high viral loads were collected during winter-spring in diverse areas where these species could be directly implied in virus overwintering [28, 33]. Proposed potential mechanisms for the interseasonal maintenance of BTV in temperate areas include: (i) virus transovarial transmission from parent to offspring insect vectors; (ii) increased duration of ruminant livestock infection; (iii) prolonged *Culicoides* adult lifespan following the active virus transmission period; (iv) ongoing and slow/low-level cycle of infection and transmission between ruminants and midges over the interseasonal period [67–69]. While we cannot exclude more mechanisms may coexist in the same area, the demonstration that *Culicoides* vectors with high viral load are found in winter-spring in conjunction with low seroconvertion rate in animals in Sardinia, would support a continuous cycle of infection and transmission between ruminants and midge vectors.

## Conclusions

Based on this study and on previous knowledge on distribution and abundance of *Culicoides* species, Bluetongue in Sardinia and probably in other Mediterranean regions, is likely to be transmitted by multiple vectors, which ensures virus spread in areas characterized by different environmental and climatic conditions. In addition, different species can be involved in virus overwintering, thus contributing to endemicity of this disease that almost continuously appeared during the last 15 years in Sardinia and the neighboring Mediterranean regions.

## Abbreviations

asl, above sea level; BT, Bluetongue; BTV, bluetongue virus; COI, mithocondrial cytochrome oxidase c subunit I; ITS2 rDNA, ribosomal internal transcribed spacer 2; MIR, minimum infection rate; RT-qPCR, Real-time Quantitative Reverse Transcription-Polymerase Chain Reaction; *s.l.*, *sensu lato*; *s.str*., *sensu stricto*
